# Early emergence of sexual dimorphism in offspring leukocyte telomere length was associated with maternal and children’s glucose metabolism—a longitudinal study

**DOI:** 10.1186/s12916-022-02687-5

**Published:** 2022-12-20

**Authors:** Kwun Kiu Wong, Feifei Cheng, Cadmon K. P. Lim, Claudia H. T. Tam, Greg Tutino, Lai Yuk Yuen, Chi Chiu Wang, Yong Hou, Michael H. M. Chan, Chung Shun Ho, Mugdha V. Joglekar, Anandwardhan A. Hardikar, Alicia J. Jenkins, Boyd E. Metzger, William L. Lowe, Wing Hung Tam, Ronald C. W. Ma

**Affiliations:** 1 Department of Medicine and Therapeutics, The Chinese University of Hong Kong, Shatin, Hong Kong; 2 Department of Obstetrics and Gynaecology, The Chinese University of Hong Kong, Shatin, Hong Kong; 3 School of Biomedical Sciences, The Chinese University of Hong Kong, Shatin, Hong Kong; 4 Chinese University of Hong Kong-Sichuan University Joint Laboratory in Reproductive Medicine, The Chinese University of Hong Kong, Shatin, Hong Kong; 5 Li Ka Shing Institute of Health Sciences, The Chinese University of Hong Kong, Shatin, Hong Kong; 6 Department of Chemical Pathology, The Chinese University of Hong Kong, Shatin, Hong Kong; 7 Diabetes and Islet Biology Group, School of Medicine, Western Sydney University, Campbelltown, Australia; 8 NHMRC Clinical Trial Centre, Faculty of Medicine and Health, University of Sydney, Camperdown, Australia; 9 Northwestern University Feinberg School of Medicine, Chicago, USA; 10 Hong Kong Institute of Diabetes and Obesity, The Chinese University of Hong Kong, Shatin, Hong Kong; 11 Chinese University of Hong Kong-Shanghai Jiao Tong University Joint Research Centre in Diabetes Genomics and Precision Medicine, Shatin, Hong Kong; 12 Department of Medicine and Therapeutics, The Chinese University of Hong Kong, Prince of Wales Hospital, Shatin, New Territories Hong Kong, China

**Keywords:** Early programming, Glucose, Telomere, Sexual dimorphism, Longitudinal study

## Abstract

**Background:**

Leukocyte telomere length (LTL) is suggested to be a biomarker of biological age and reported to be associated with metabolic diseases such as type 2 diabetes. Glucose metabolic traits including glucose and insulin levels have been reported to be associated with LTL in adulthood. However, there is relatively little research focusing on children’s LTL and the association with prenatal exposures. This study investigates the relationship between maternal and offspring glucose metabolism with offspring LTL in early life.

**Methods:**

This study included 882 mother-child pairs from the HAPO Hong Kong Field Centre, with children evaluated at age 7.0 ± 0.4 (mean ± SD) years. Glucose metabolic traits including maternal post-load glucose during pregnancy, children’s glucose and insulin levels, and their derived indices at follow-up were measured or calculated. Offspring LTL was assessed using real-time polymerase chain reaction.

**Results:**

Sex- and age-adjusted children’s LTL was found to be associated with children’s HOMA-IR (*β*=−0.046 ± 0.016, *p*=0.005). Interestingly, both children’s and maternal post-load glucose levels were positively associated with children’s LTL. However, negative associations were observed between children’s LTL and children’s OGTT insulin levels. In addition, the LTL in females was more strongly associated with pancreatic beta-cell function whilst LTL in males was more strongly associated with OGTT glucose levels.

**Conclusions:**

Our findings suggest a close association between maternal and offspring glucose metabolic traits with early life LTL, with the offspring sex as an important modifier of the disparate relationships in insulin production and response.

**Supplementary Information:**

The online version contains supplementary material available at 10.1186/s12916-022-02687-5.

## Background

Leukocyte telomere length (LTL) is associated with adverse outcomes and metabolic diseases in adults. In adulthood, shorter LTL has been reported to be associated with higher fasting glucose [1], HbA1c [2], postprandial glucose [3] and HOMA-IR [4, 5]. However, the number of studies on telomere length in children is relatively limited. LTL at younger ages was reported to be related to several factors, including obesity [6], pollution [7] and socioeconomic status [8]. One important reason to study LTL at an early age is the rapid change in LTL that occurs only at early ages [9, 10]. Whilst it is common to observe shortening of <100 basepairs per year in adults, shortening over 1000 basepairs per year has been reported in the first 4 years after birth [9]. This difference in changes in telomere length highlights the importance of studying LTL at an early age.

LTL is highly correlated between early and later life [10]. This supports LTL may be involved in the Developmental Origins of Health and Disease (DOHaD) hypothesis, which was first proposed to explain the association between early life exposures and the risks of diseases in later life. One question that the DOHaD hypothesis poses is whether the associations between LTL and many later-life cardiovascular traits also exist earlier in life. Whilst some studies have shown associations between adulthood LTL and BMI [11], glucose metabolism [1] and lipid profile [12], the relationships between LTL and these traits in children have not been well-researched. We hypothesized that offspring LTL may be associated with children’s metabolic traits and may exhibit a relationship with maternal exposures. To address this, we examined the relationship between offspring LTL and maternal and offspring glucose metabolic traits in mother-offspring pairs from a well-phenotyped cohort

## Methods

### Subjects

The Hyperglycemia and Adverse Pregnancy Outcome (HAPO) Study was an observational multi-centre international study aiming to examine the risk of adverse outcomes during pregnancy, in association with maternal glucose levels below those diagnostics of diabetes mellitus. Details of this cohort have been published [13]. Our current study included only mother-offspring pairs recruited from the Hong Kong Field Centre [14]. In this study, subjects were all of Southern Han Chinese ancestry living in Hong Kong. A total of 1683 mothers with singleton pregnancies were recruited in the Hong Kong field centre at the Prince of Wales Hospital from 2000 to 2006 (exclusion criteria are listed in the supplementary information). At ~28 weeks gestation, eligible participants underwent a 75-g standard oral glucose tolerance test (OGTT) with fasting, 1-h and 2-h glucose levels and completed standardized questionnaires for prenatal data. Anthropometric measurements were also obtained. As a safety measure, an additional maternal blood sample was also collected between 34 and 37 weeks of gestation for measuring of blood plasma glucose level (PG) to identify potential hyperglycaemia above a set threshold that would require unblinding.

#### Recruitment at follow-up

Subjects were called back for follow-up at postnatal year 7 at the same hospital from 2009 to 2013. Detailed documentation of their background including family history of diabetes mellitus, maternal smoking and drinking habits at the follow-up study, and children’s lifestyle factors such as the frequency of physical activity, were recorded using standardized questionnaires. Some participants could not be contacted or declined to participate in the follow-up study, but of the original 1683 HAPO Hong Kong participants, 970 mother-child pairs attended the follow-up visit. After the exclusion of preterm offspring (<37weeks gestation), 926 offspring were included in this current study (Supplementary figure 1). Assuming type I and type II error rates as *α*=0.05 and *β*=0.2, respectively, an estimated sample size of 229 would be needed to identify LTL association with insulin resistance based on previously published data [5]. For a similar set of estimated parameters, *n*=716 was adequate to identify the association between childhood BMI and LTL in 8-year-olds [15]. Our study was an established cohort and the number of available samples was limited by the numbers recruited. Maternal GDM was retrospectively diagnosed using IADPSG definition, i.e. fasting PG≥ 5.1 mmol/l, 1-h PG≥ 10.0 mmol/l, and/or 2-h PG≥8.5 mmol/l during the 75g OGTT.

### Laboratory measurements

PG at follow-up was measured with the hexokinase method using an automated analyser. Plasma insulin and C-peptide levels were measured using an immunoassay analyser. The lower limits of detection were 2.0 mIU/L and 0.1 mg/L, respectively. For maternal blood drawn at the prenatal visit, serum HbA1c and PG were measured at the HAPO Central Laboratory [13]. For offspring at follow-up, plasma glucose and insulin levels were measured at multiple time-points (0, 15, 30, 60, 120min) during the OGTT, as described earlier [14].

#### LTL measurement

Newborn DNA was isolated from the umbilical cord blood collected within 5 min of delivery. DNA was extracted by the Centre for Genetic Medicine of Northwestern University Feinberg School of Medicine using the Gentra Puregene Blood kit (Qiagen, Hilden, Germany) and stored at −80°C. Frozen DNA samples were transported between the USA and Hong Kong on dry ice with temperature monitoring to minimize degradation. Offspring whole blood collected at the follow-up visit was stored in a 10-ml EDTA blood tube, and DNA was extracted with the standard phenol-chloroform method. The DNA pellet, after repeated alcohol washes, was re-suspended with high-quality qPCR grade nuclease-free water and stored for later use. The protocol for LTL measurement with the application of real time-qPCR was a slight modification of the protocol of Cawthon [16]. Our protocol was refined and optimized by collaborators [17]. This protocol was validated in earlier studies [18]. Details for the current real-time measurement of PCR LTL are available in the supplementary section and the published protocol [17]. In brief, Ct values were obtained by measuring telomere and a single-copy gene. By calculating the delta-delta Ct values, a telomere-single-copy gene ratio (T/S ratio) could be calculated to obtain relative telomere length values for analysis. A higher delta-delta Ct value suggests a longer telomere length of a sample. Cord blood and children’s LTL from the same individual were measured on the same plate to minimize plate-to-plate variation.

### Statistical analysis

Data are presented as mean±SD, median (Q1, Q3), or percentage (%). Data with skewness within ±1 were considered to be normally distributed. Otherwise, data were transformed for linear regression analyses. A constant would be added for negative values. Comparisons between groups were performed with Student’s *t*-test. Chi-square (χ2) or Fisher’s exact tests were used, as appropriate, for categorical variables. Bivariate linear regression was conducted to test the relationships between LTL and baseline characteristics. Due to minimal missing data, we did not perform any data imputation. In order to check the sensitivity of the associations, tertiles were also examined.

Given the total number of glucose metabolic traits being examined (*N*=15) and the inter-relatedness of many traits, we divided the 15 traits into 4 groups: (1) glucose traits (fasting glucose, 1-h, 2-h, glucose area under the curve (GAUC)); (2) insulin traits (fasting insulin, 1-h, 2-h, insulin area under the curve (IAUC)); (3) beta-cell function (fasting C-peptide, homeostasis model assessment of beta-cell function (HOMA-BCF), insulinogenic index, oral disposition index, beta-cell function derived from IAUC/ GAUC (BCF)); (4) insulin resistance/sensitivity (HOMA-IR and Matsuda index). Therefore, we used an arbitrary *p*-value of 0.05/4=0.0125 as indicative of suggestive association, instead of 0.05/15, i.e. *p*<0.003 as the threshold of applying a standard Bonferroni correction for testing of multiple traits. We have included a heatmap to show the inter-relatedness of the 15 traits (supplementary figure 2).

The formulas used to calculate the different glucose- and insulin-derived indices are included in the supplementary information. The inverse normal transformation was applied to traits that were heavily skewed, such as insulin levels from the OGTTs. They were transformed to approximate normality for the linear regression model analyses. The traits were first adjusted for age and sex in model 1, or age alone after stratification by sex. The fully adjusted model included adjustment for maternal factors/variables, such as maternal age, and children’s variables, such as children’s BMI. The resulting residuals were transformed to a *z*-score (i.e. *i*th residual-mean (residual)/SD (residual)).

The percentage change in LTL over time for each subject was calculated using the formula: [(children’s LTL–cord blood LTL)/cord blood LTL] × 100% as a robust mean to explore the association of LTL with measures of pancreatic function. This was first done by performing absolute telomere conversion with the application of whole-genome sequencing (WGS) data using Telseq, which we used previously to estimate absolute LTL in other cohorts [19, 20]. Subjects were categorized into 3 different groups: “shortened” (LTL change <−10%), “maintained” (10%>LTL change>−10%), “lengthened” (LTL change >10%) with reference to previous studies [21, 22]. Data analyses were performed with the Statistical Package for the Social Sciences, version 25.0 (SPSS Inc., Chicago, USA), and R version 4.0.2 (R Core Team, Vienna, Austria). *P*≤0.05 (two-tailed) was considered statistically significant.

## Results

The clinical characteristics of the subjects in the Hong Kong HAPO follow-up study are described in Table 1. Of the 926 subjects included, 882 children at age 7.0±0.4 years had measured LTL as well as glucose- and insulin-related traits. Among the children, 48% were female. A subset of 313 offspring had cord blood DNA collected. The overall inter-plate coefficient variations of the telomere and HBG assays were 1.34% and 0.48%. The overall intra-plate CV was 0.93% for telomere and 0.73% for HBG.

**Table 1:** Basic characteristics for mothers and offspring at follow-up stratified by children’s LTL tertiles

	Tertile 1 (***n***=294)	Tertile 2 (***n***=294)	Tertile 3 (***n***=294)	Total (***n***=882)	
Children’s LTL	0.97 ± 0.28	1.50 ± 0.11	2.00 ± 0.26	1.49 ± 0.48	
Cord blood LTL	1.42 ± 0.52	1.65 ± 0.52	2.05 ± 0.61	1.74 ± 0.62	**<0.001**
**Maternal**	**T1**	**T2**	**T3**	**Total**	**P**
Maternal age EDC (yr)	30.7 ± 4.9	31.4 ± 4.4	31.8 ± 4.5	31.3 ± 4.6	**<0.001**
Pre-preg BMI (kg/m^2^)	20.9 ± 2.8	21.0 ± 3.0	20.8 ± 2.8	20.9 ± 2.9	0.569
Prenatal smoking ^#^	6 (2.0)	5 (1.7)	2 (0.7)	13 (1.5)	0.363
Parity, null vs ≥ 1, null^#^	179 (60.9)	178 (60.5)	159 (54.1)	516 (58.5)	0.346
Maternal current DM^#^	25 (8.5)	36 (12.2)	37 (12.6)	98 (11.1)	0.214
Paternal current DM^#^	31 (10.5)	32 (10.9)	31 (10.5)	94 (10.7)	0.989
C-section^#^	59 (20.1)	79 (26.9)	73 (24.8)	211 (23.9)	0.140
Breastfeeding^#^	139 (47.3)	157 (53.4)	142 (48.3)	438 (49.7)	0.312
Birthweight (g)	3227 ± 358	3238 ± 402	3202 ± 407	3220 ± 392	0.516
Maternal FBG (mmol/L)	4.3 ± 0.3	4.4 ± 0.3	4.4 ± 0.3	4.4 ± 0.3	0.059
Maternal OGTT GAUC	12.9 ± 1.9	13.3 ± 2.3	13.3 ± 2.1	13.2 ± 2.2	**0.043**
**Children**	**T1**	**T2**	**T3**	**Total**	***p***
Follow-up age (year)	7.0 ± 0.4	7.0 ± 0.4	7.0 ± 0.5	7.0 ± 0.4	0.495
Sex (female %)^#^	124 (42.2)	134 (45.6)	163 (55.4)	421 (47.7)	**0.004**
BMI (kg/m2)	15.2 ± 2.5	15.2 ± 2.3	14.8 ± 2.0	15.1 ± 2.3	**0.027**
Children’s exercise levels (0, 1, 2)					**0.003**
0	20 (6.8)	31 (10.5)	38 (12.9)	89 (10.1)	
1	155 (52.7)	150 (51.0)	162 (55.1)	467 (52.9)	
2	119 (40.5)	134 (45.6)	94 (32.0)	347 (39.3)	

^#^Shown in number (percentage). *Shown with median [Q1–Q3] PLUS *p*-values comparison with natural log transformation in correlation test. *P* shown at the last column compare for the trend between T1, T2 and T3 only

### Relationship between children’s LTL and maternal and offspring baseline factors

In the unadjusted linear regression model, children’s LTL was positively associated with maternal age (*β*=0.013±0.003, *p*<0.001). Offspring exercise level was negatively associated with children’s LTL (*β*=−0.022±0.007, *p*<0.001). Offspring sex was significantly associated with LTL, with female offspring having longer LTL (*β*=0.116±0.032, *p*<0.001). Offspring BMI (*β*=−0.016±0.007, *p*=0.02) was negatively associated with LTL (Supplementary table 1).

### Relationship between children’s LTL and offspring glucose metabolic traits

Children’s LTL showed associations with offspring OGTT glucose and insulin levels (Supplementary table 2). In an unadjusted model, offspring OGTT glucose levels at 1 h (*β*=0.028±0.011, *p*=0.009) were positively associated with LTL, whilst 2-h glucose levels were positively associated (*β*=0.06±0.017, *p*<0.001) in a fully adjusted model. GAUC (*β*=0.05, *p*=0.003) was also positively associated with children’s LTL, but fasting glucose was not associated in any model (*β*=−0.065±0.045, *p*=0.146). Fasting C-peptide, which reflects insulin secretion, was negatively and suggestively associated with offspring LTL after adjustment for sex and age (*β*=−0.054, *p*=0.001).

HOMA-IR, which estimates insulin resistance, was negatively associated with LTL after adjustment for sex and age (*β*=−0.046, *p*=0.005) and remained marginally associated after full adjustment. Conversely, the Matsuda index, a measure of insulin sensitivity, demonstrated a suggestive positive association with LTL after adjusting for sex and age (*β*=0.038, *p*=0.022). For measures of pancreatic beta-cell function, HOMA-BCF was negatively associated with children’s LTL after adjusting for sex and age (*β*=−0.042, *p*=0.011), but the association was attenuated after adjustment for parental factors. HOMA-BCF was negatively associated after adjustment for sex and age-adjusted LTL (*β*=−0.042, *p*=0.011). The oral disposition index (oDI) was not associated with children’s LTL in any of the models. Groups stratified according to children’s LTL tertiles suggested a clear linear trend between LTL and fasting insulin, fasting C-peptide, insulinogenic index, beta-cell function and HOMA-IR (Supplementary table 3). Children in the tertile with the shortest telomere length had significantly higher C-peptide and HOMA-IR.

### Relationship between offspring LTL and maternal glucose traits during pregnancy

There were positive associations between children’s LTL and maternal glucose levels at time points following a glucose load in the unadjusted analysis (Table 2). Offspring LTL was positively associated with unadjusted maternal 2-h glucose (*β*=0.032±0.016, *p*=0.008) and GAUC values (*β*=0.019±0.007, *p*=0.01); both traits demonstrated suggestive associations with children’s LTL after further adjustments. The association between children’s LTL and maternal fasting blood glucose was significant before adjusting for children’s and maternal confounders (*β*=0.042, *p*=0.009). There was a trend towards an association between GDM (diagnosed according to IADPSG criteria) and children’s LTL (*β*=0.085±0.046, *p*=0.063), suggesting foetal exposure to GDM may be associated with longer offspring LTL. In short, maternal glucose levels appeared to be positively associated with children’s LTL, and this association remained significant after adjusting for children’s postnatal factors. This association however was attenuated after adjustment for maternal variables. Examination of cord blood LTL with the newborn characteristics of interest (Supplementary table 4) demonstrated a negative association between maternal fasting glucose and cord blood LTL after full adjustment (*β*=−0.087, *p*=0.01).

**Table 2:** Associations between children’s LTL with maternal and newborn characteristics

	Model 0			Model 1			Model 2			Model 3		
	Beta	SE	***P***	Beta	SE	***P***	Beta	SE	***P***	Beta	SE	***P***
Maternal HbA1c (%)	−0.061	0.047	0.191	−0.018	0.017	0.272	−0.017	0.017	0.307	−0.03	0.017	0.08
Maternal FBG (mmol/L)	0.121	0.048	**0.012**	0.046	0.016	**0.004**	0.042	0.016	**0.009**	0.034	0.016	0.035
Maternal glucose 60 (mmol/L)	0.021	0.01	0.035	0.031	0.016	0.058	0.028	0.016	0.08	0.017	0.016	0.29
Maternal glucose 120 (mmol/L)	0.032	0.012	**0.008**	0.038	0.016	0.018	0.036	0.016	0.025	0.026	0.016	0.106
Maternal OGTT GAUC	0.019	0.007	**0.01**	0.035	0.016	0.028	0.034	0.016	0.037	0.023	0.016	0.151
Maternal GDM (IADPSG) (%)	0.085	0.046	0.063	−0.025	0.016	0.113	−0.016	0.016	0.331	−0.018	0.016	0.255

Model 0: unadjusted; Model 1 (basic): adjusted for children’s sex and age

Model 2 (perinatal and children’s environmental factors): Model 1 + C-section or not, history of breast feeding, children’s BMI and exercise level (0, 1, 2)

Model 3 (parental and newborn effects): Model 2 + maternal prepregnant BMI, maternal age at EDC, parity, birthweight and current mother and father diabetes or not

### Relationship between children’s LTL and offspring pancreatic and maternal glycaemic traits stratified by sex

At the 7-year follow-up of HAPO mother-offspring pairs, we noted different relationships between maternal glucose and offspring phenotype according to offspring sex [14]. Differences in metabolic traits between boys and girls were evident (Supplementary table 5). BMI was different between boys and girls. Males had higher glucose and insulin during fasting, whilst female offspring had higher glucose and insulin levels following a glucose load. HOMA-BCF was significantly higher in female offspring whilst HOMA-IR was higher in male offspring. These comparisons suggested differences in metabolic traits between male and female offspring, highlighting the need for sex-stratified analyses.

Primary analyses suggest female children had longer telomere length, with a mean TL difference of 0.13 delta-delta Ct value (Supplementary table 5). For male offspring, significantly positive relationships were observed between LTL and 1-h (*β*=0.067, *p*=0.005) and 2-h glucose levels (*β*=0.075, *p*=0.002) and GAUC (*β*=0.072, *p*=0.002) after full adjustment (Table 3). In contrast, LTL in female offspring was closely associated with insulin levels at different OGTT time points after adjustment for age. Insulin levels at different time-points were significantly and negatively associated with female LTL after full adjustment. Fasting C-peptide was also negatively associated with female LTL (*β*=−0.064, *p*=0.007) after adjustment for age. Insulinogenic index (*β*=−0.06, *p*=0.012) in the fully adjusted model and beta-cell function (*β*=−0.065, *p*=0.007) after adjustment for age were negatively related with female LTL whilst Matsuda index (*β*=0.073, *p*=0.002) was positively associated with female LTL. HOMA-IR was also marginally and negatively associated with female LTL after the fully adjusted model.

**Table 3:** Associations of sex-stratified offspring LTL at 7 with glucose and insulin relating traits

	Model 0						Model 1				Model 2			
	Male			Female			Male		Female		Male		Female	
	Beta	SE	***P***	Beta	SE	***P***	Beta	***P***	Beta	***P***	Beta	***P***	Beta	***P***
Fasting glucose	−0.018	0.067	0.8	−0.051	0.064	0.43	−0.005	0.838	−0.023	0.335	0.001	0.966	−0.039	0.101
1-h glucose	0.042	0.016	**0.01**	0.015	0.015	0.32	0.066	**0.005**	0.02	0.388	0.067	**0.005**	0.005	0.839
2-h glucose	0.087	0.025	**0.001**	0.038	0.024	0.11	0.074	**0.002**	0.039	0.096	0.075	**0.002**	0.037	0.114
AUCglu at 0–120 min	0.001	0.0002	**0.002**	0.000286	0.00019	0.14	0.073	**0.002**	0.033	0.162	0.072	**0.002**	0.023	0.331
Fasting Insulin	0.003	0.004	0.42	−0.014	0.006	0.03	−0.012	0.612	−0.047	0.044	0.007	0.769	−0.051	0.032
1 h insulin	−0.0004	0.001	0.71	−0.001	0.001	0.16	−0.001	0.98	−0.055	0.021	0.001	0.956	−0.056	0.019
2 h insulin	0.001	0.001	0.45	−0.002	0.001	0.04	0.046	0.051	−0.07	**0.003**	0.054	0.023	−0.048	0.043
AUCins at 0–120 min	−4E−06	0.00001	0.67	−1.571E−05	9E−06	0.08	0.009	0.694	−0.056	0.02	0.015	0.518	−0.057	0.018
Fasting C-pep	−9.40E−02	5.40E−02	0.08	−0.153	0.061	**0.01**	−0.041	0.082	−0.064	**0.007**	−0.025	0.286	−0.053	0.026
HOMA-BCF	−0.001	0.00036	0.14	−0.001	0.0003	0.07	−0.022	0.363	−0.037	0.118	−0.01	0.677	−0.027	0.255
Insulinogenic index 30	−0.00023	0.00024	0.34	−0.001	0.0003	**0.01**	−0.022	0.367	−0.07	**0.003**	−0.008	0.734	−0.06	**0.012**
Disposition Index	0.001	0.002	0.78	−0.005	0.003	0.16	−0.019	0.437	−0.038	0.107	−0.016	0.496	−0.026	0.282
Beta-Cell function	−0.001	0.001	0.32	−0.003	0.001	**0.01**	−0.004	0.869	−0.065	**0.007**	0.003	0.897	−0.059	0.014
HOMA-IR	−0.059	0.031	0.06	−0.062	0.03	0.04	−0.033	0.159	−0.051	0.029	−0.017	0.469	−0.052	0.027
Matsuda Index	0.000272	0.003	0.92	0.005	0.003	0.04	0.004	0.877	0.063	**0.008**	−0.032	0.184	0.073	**0.002**

Model 0: unadjusted; Model 1 (basic): adjusted for children’s age; Model 2 (parental effects, perinatal and children’s environmental factors): Model 1 + maternal prepregnant BMI, maternal age at EDC, maternal AUCglu during pregnancy, parity; further adjusted for current maternal hypertensive status for blood pressure; or further adjusted for current maternal and paternal diabetes status for glucose and insulin levels, as well as indices for β cell function and insulin sensitivity, C-section or not, gestational age at delivery, history of breast feeding, birthweight, children’s BMI and exercise level (0, 1, 2). The values in model 1 and 2 subjected to normal inverse transformation; their SEs were all 0.023–0.024 and were ignored from the table. The unit for glucose, insulin and C-peptide are mmol/l, mIUL and ug/L respectively

### Relationship between LTL percentage change and maternal and offspring traits

By combining LTL measurements from cord blood and children, the association between the change in LTL and different metabolic traits was examined. There were 308 subjects with both measurements of cord blood and children’s LTL. LTL was longer in 15% of offspring compared to their cord blood LTL, whilst the LTL was shorter in 36% (Supplementary figure 3). Offspring in the “longer LTL” group (Supplementary table 6) had lower IAUC, insulinogenic index, disposition index, beta-cell function, and HOMA-IR. This group also had a higher Matsuda index, reflecting their higher overall insulin sensitivity. Similar trends were also observed for other glucose metabolic traits, except the oral disposition index. No significant difference was observed in the maternal pregnancy traits among the groups; however, all maternal glucose traits were insignificantly higher in the “longer LTL” group, which also had a numerically higher percentage of untreated maternal GDM, though this was not statistically significant.

## Discussion

In this 7-year longitudinal study of mother-offspring dyads, we observed the following: (1) Children’s LTL was associated with glucose metabolic traits at age 7 years. (2) Children’s LTL and cord blood LTL were associated with maternal glycaemic traits. (3) Sex was a key determinant in the associations between LTL and glucose-related traits, with insulin levels and related indices reflecting glucose metabolic functions demonstrating associations in female offspring, whilst associations of LTL with post-load glucose levels were observed in male offspring. (4) By stratifying telomere length change from newborn to follow-up, considering the individual variance on cord blood baseline LTL, similar associations between glucose metabolic traits across LTL change groups were shown. These results highlighted a close association between offspring LTL and glucose metabolic traits in early life.

A significant difference in telomere length between males and females was observed, with female offspring having longer LTL than males, similar to other reports, and clear differences in associations with metabolic traits were also observed. Sex differences in telomere length have been observed in earlier studies [23–25] and are discussed below. Children’s LTL was negatively associated with children’s BMI, similar to earlier studies [15, 26]. For glucose metabolic traits, HOMA-IR, which estimates insulin resistance, was significantly and negatively associated with LTL in other studies in adults [2, 5]. One study, which tried to predict diabetes progression with baseline LTL in 108 Chinese adults, reported a positive association between adulthood LTL and the Matsuda Index [27], which provides a measure of whole-body insulin sensitivity [28]. A Danish cohort consisting of teenage offspring of mothers with or without GDM also reported a negative association between LTL in 9- to 16-year-old females and their HOMA-IR and fasting insulin levels [23]. In our study, negative associations were observed between female LTL and fasting insulin levels and HOMA-IR as well as positive associations between LTL and Matsuda index, both overall and in females. Many of our findings are consistent with reports from earlier studies. However, unlike several previous studies which reported an association of longer adult LTL with lower fasting insulin and fasting and post-load glucose [1, 5], we have observed different relationships: significant associations of longer offspring LTL with lower insulin release and higher glucose following a glucose load. As estimated by both HOMA-BCF and the insulinogenic index-30, pancreatic beta-cell function was negatively associated with children’s LTL. This appears to give rise to conflicting results, i.e. that longer LTL, on the one hand, was associated with better whole-body insulin sensitivity and lower insulin resistance, whilst also being associated with poorer pancreatic beta-cell function and lower insulin release and higher glucose.

### Relationship between childhood insulin sensitivity and LTL

One key factor in the progression of diabetes is reduced insulin sensitivity, which usually occurs in late adulthood [29]. In many reports, T2DM patients were reported as having shorter LTL than healthy counterparts [2]. It is possible that the child with larger body size and adiposity, as shown with the negative association between children’s BMI and their LTL, has increased the whole-body insulin resistance. This, however, was compensated for by an increase in pancreatic beta-cell insulin production. Such compensation may not occur in aged adults with pancreatic beta-cells exposed to prolonged metabolic challenge, leading to pancreatic beta-cell failure due to inadequate beta-cell compensation [30], resulting in hyperglycaemia and diabetes. Considering the pancreas is still actively growing and increasing in volume at age seven [31], a large proportion of their pancreatic beta-cells would be relatively young [32]. The beta-cell could therefore still compensate for the decreased insulin sensitivity by increasing insulin output, especially given that our studies were conducted in a relatively healthy cohort. The LTL change results suggested something similar. The tendency of hyperinsulinemia in the youths, when compared with similar levels of obesity and dysglyceamia in adults, may suggest that the greater amount of insulin released may not represent more “robust” pancreatic function. Instead, the greater workload in the endocrine pancreas or the possible increased insulin release to compensate for the decreased insulin-independent glucose uptake could represent an early sign of beta-cell dysfunction. This observation may be in line with our results between LTL, glucose and insulin levels [33]. The shorter LTL group comprised of offspring with lower Matsuda index and higher HOMA-IR, and at the same time, these offspring were observed to have greater insulin release during an OGTT and higher insulinogenic index and beta-cell function. Both increased cellular oxidative stress [34] and active pancreatic cell division, which are key factors interacting with the telomere [12], may have contributed to the shorter LTL. The fact that oral disposition index (oDI), the only dynamic measure of beta-cell function which controls for prevailing insulin resistance, was not associated with children’s LTL, despite the many associations between pancreatic traits and children’s LTL, may suggest the associations of LTL with beta-cell function were perhaps driven by the association between LTL and insulin resistance. Several reports have observed that LTL may be associated differently with traits in children versus in adulthood [35–37]. This and our observations highlight the dynamics of LTL across life stages and stress the importance of studies of LTL at an early age.

### Maternal pregnancy glucose level in association with offspring LTL

There were limited relationships between cord blood LTL and maternal glucose traits. The observation that appeared consistent in our dataset, however, was an insignificant positive association between maternal post-load glucose levels during pregnancy and cord blood LTL. The same positive associations were also found with children’s LTL. We hypothesized that the lower post-load glucose levels found in children with shorter LTL were the result from the compensatory insulin secretion by children’s pancreas due to the prevailing insulin resistance. It is possible that maternal glucose levels during pregnancy (within a certain physiological range) may have affected the threshold for the pancreas to respond with compensatory insulin release or pancreatic cell growth. LTL was related to both maternal and offspring glucose in the same direction. It is conceivable that LTL may be involved in such glucose regulatory action as illustrated schematically in Supplementary figure 4. Our findings for the associations between both maternal and children’s glucose levels and children’s LTL are potentially in line with findings from the HAPO Follow-up Study: higher maternal glucose during pregnancy leads to higher childhood glucose in the offspring [38]. Similar positive correlations were also found in our cohort (supplementary figure 5).

### Sexual dimorphism in associations between childhood pancreatic traits and LTL

**Fig. 1:**
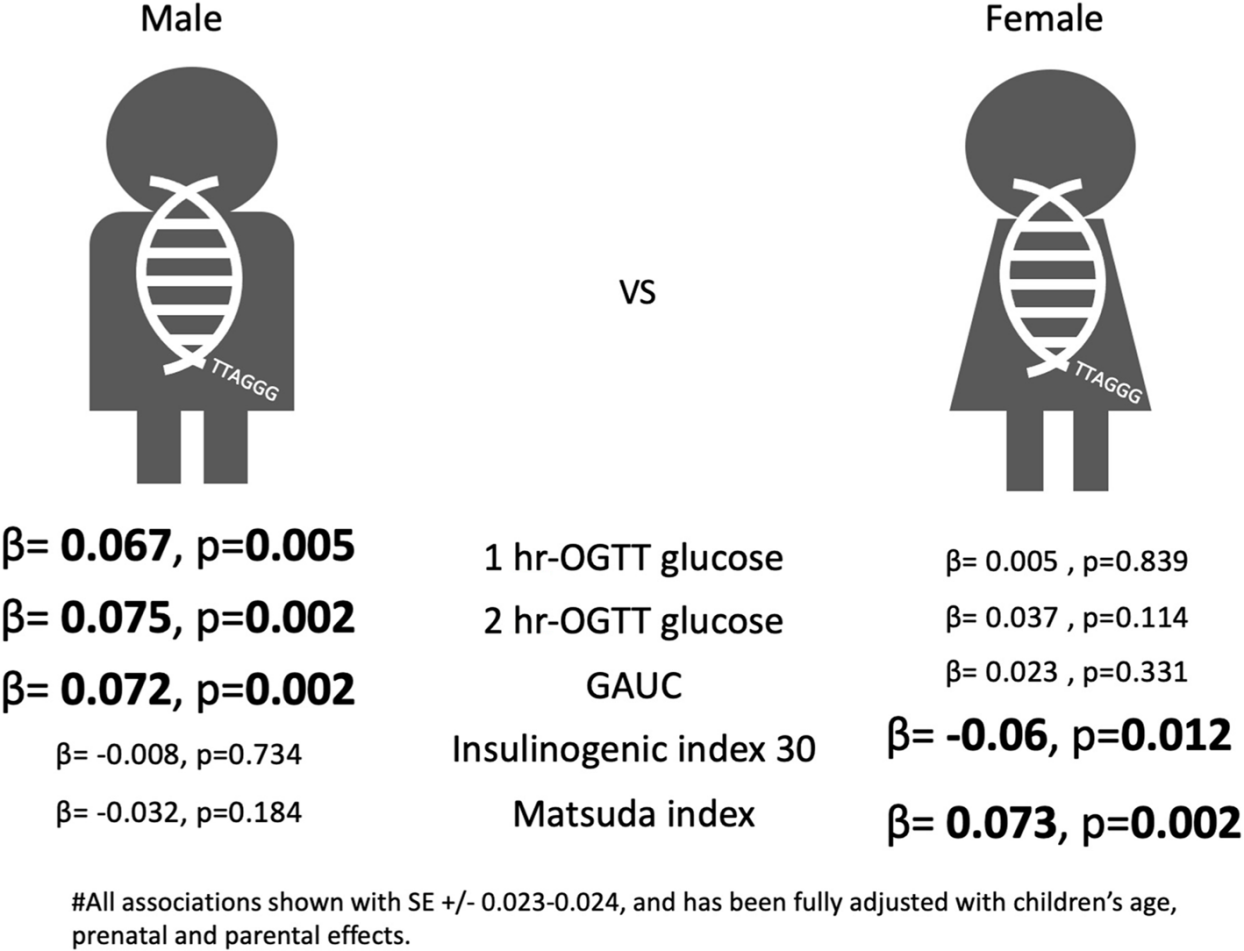
Sexual dimorphism between the association of children’s LTL and glucose metabolic traits

We followed other early-life LTL studies [23, 39] and explored the LTL associations stratified by sex (Fig. 1). The female children’s LTL associations with measures of pancreatic function were stronger than those in males, whilst male children’s LTL was strongly associated with glucose levels following a glucose load. A limited but similar sex-stratified difference was also observed for the association of maternal post-load glucose levels with both cord blood and children’s LTL stratified by sex (Supplementary table 7 and 8). Female LTL was more strongly associated with maternal OGTT glucose levels whilst male LTL was not associated. In our previous analysis [14], male offspring exposed to maternal hyperglycaemia were more strongly associated with glucose intolerance in children, whilst female offspring exposed to maternal hyperglycaemia were more strongly associated with children’s adiposity. Differences in pregnancy survival strategies could explain this: male foetuses tend to grow faster and bigger whilst female foetuses tend to invest more in the placenta and allocate more reserve on fat masses [40]. Such differences in survival strategies by sex may result in differences in postnatal effects at an early age and later in life. Sex-specific associations were also observed in a previous study of telomere length at age 9–16 years in a Danish cohort [22]. LTL in females was more strongly associated with HOMA-IR and fasting insulin whilst LTL in males was more strongly associated with high-sensitivity C-reactive protein, an inflammatory marker. There were also reports on sex-specific differences in adult glycaemic control and pancreatic function [41], such as females being more likely to suffer from glucose intolerance whilst males were more likely to have elevated fasting glucose. The associations between children’s LTL and glucose metabolism-related traits may be subject to both differences in in utero growth, sex-specific physiology and the many sex-specific genomic and epigenomic changes [42]. The underlying mechanisms are unclear and warrant further mechanistic studies to advance understanding in this area.

### Strengths and limitations

There were several limitations in our study. Although the HAPO cohort is well-characterized, some traits previously reported to affect LTL are not available in this study, such as socioeconomic status and paternal age [8], as well as maternal LTL [21]. It was not possible to eradicate the potential of unaccounted for confounders that could modify the associations between offspring LTL and glucose metabolism. Another limitation is the inability to conclude causality. We could only speculate, for example, whether increased insulin resistance drove pancreatic compensation. It may be possible in the future to include methods such as Mendelian randomization (MR) for further mechanistic studies and thereby obtain a better understanding of the causal links. Such MR studies would provide additional insights, as highlighted by some LTL studies incorporating this approach [43, 44]. We also acknowledge that real-time qPCR-based methods of measuring telomere length in general had larger measurement errors compared to terminal restriction fragment (TRF) length by Southern blot, and hence, the latter may be able to detect a more subtle association between LTL and traits [12]. Nevertheless, we believe our findings provide some novel insights. We observed not only a close association between glucose metabolism and LTL during pregnancy and childhood but also longitudinal associations between telomere length and metabolic traits. More research in this area is warranted to better understand these relationships and further define the relationship between LTL and the developmental origins of adult health and disease.

## Conclusions

We have observed children’s and cord blood LTL were both associated with maternal and offspring childhood glycaemic traits, and sex was observed to be a key determinant of the associations.

## Supplementary Information

**Additional file 1.**

**Additional file 2: Supplementary table 1.** Univariate associations between cord and children’s LTL with their basic characteristics. **Supplementary table 2.** Offspring LTL at follow up and associations with glucose and insulin related traits. **Supplementary table 3.** Basic characteristics offspring at follow-up traits stratified according to children’s LTL tertiles. **Supplementary table 4.** Regression result for Cord blood LTL association with newborn and maternal glycaemic traits. **Supplementary table 5.** Sex-stratified maternal and offspring characteristics. **Supplementary table 6.** Glucose and insulin traits stratified according to LTL change group. **Supplementary table 7.** Sex -stratified children’s LTL associations with maternal and newborn characteristics. **Supplementary table 8.** Sex -stratified cord blood LTL associations with maternal and newborn characteristics. **Supplementary table 9.** Baseline characteristics between mothers returned for follow-up to mothers without follow-up. **Supplementary figure 1.** Diagram for HAPO and HAPO follow-up recruitment and mother-child pair excluded with reasons. **Supplementary figure 2.** The Heatmap showing the correlations between maternal (MA) and offspring (OS) glucose and insulin traits. **Supplementary figure 3.** Telomere length percentage change histogram. **Supplementary figure 4.** Schematic illustration on the conceptual associations between glucose and LTL. **Supplementary figure 5.** Correlations between maternal (MA) and offspring (OS) glucose levels. **Supplementary figure 6.** The calculation of glucose area under the curve.

## Data Availability

The datasets generated during and/or analysed during the current study are available from the corresponding author on reasonable request.

## Abbreviations

BMI: Body mass index
DOHaD: Developmental Origins of Health and Disease
GAUC: Glucose area under the curve
HbA1c: Glycated haemoglobin
HOMA-BCF: Homeostasis model assessment of beta-cell function
HOMA-IR: Homeostasis model assessment of insulin resistance
HAPO: Hyperglycemia and Adverse Pregnancy Outcome Study
IAUC: Insulin area under the curve
LTL: Leukocyte telomere length
MR: Mendelian randomization
OGTT: Oral Glucose Tolerance Test
PG: Plasma glucose level
T2DM: Type 2 diabetes mellitus
