# Supplementary material for: Early emergence of sexual dimorphism in offspring leukocyte telomere length was associated with maternal and children’s glucose metabolism—a longitudinal study
Source: BMC Med. 2022 Dec 20;20:490. doi: 10.1186/s12916-022-02687-5 (PMC9764638; doi:10.1186/s12916-022-02687-5)
Supplement: Supplementary file 1 — Additional file 1. [file 12916_2022_2687_MOESM1_ESM.pdf]

Male		Female
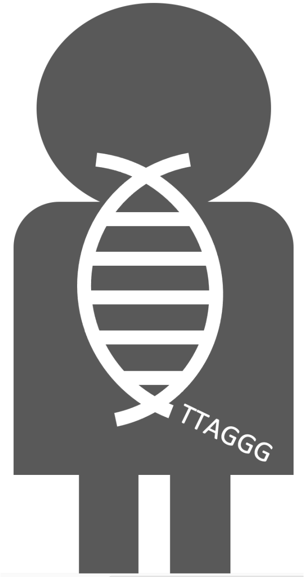	VS	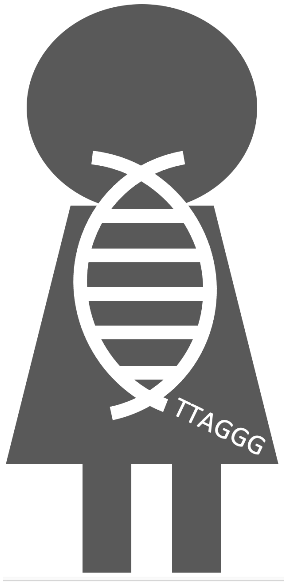
β= **0.067**, p=**0.005**	1 hr-OGTT glucose	β= 0.005 , p=0.839
β= **0.075**, p=**0.002**	2 hr-OGTT glucose	β= 0.037 , p=0.114
β= **0.072**, p=**0.002**	GAUC	β= 0.023 , p=0.331
β= -0.008, p=0.734	Insulinogenic index 30	β= **-0.06**, p=**0.012**
β= -0.032, p=0.184	Matsuda index	β= **0.073**, p=**0.002**

#All associations shown with SE +/- 0.023-0.024, and has been fully adjusted with children's age, prenatal and parental effects.
