# Supplementary material for: Early emergence of sexual dimorphism in offspring leukocyte telomere length was associated with maternal and children’s glucose metabolism—a longitudinal study
Source: BMC Med. 2022 Dec 20;20:490. doi: 10.1186/s12916-022-02687-5 (PMC9764638; doi:10.1186/s12916-022-02687-5)
Supplement: Supplementary file 2 — Additional file 2: Supplementary table 1. Univariate associations between cord and children’s LTL with their basic characteristics. Supplementary table 2. Offspring LTL at follow up and associations with glucose and insulin related traits. Supplementary table 3. Basic characteristics offspring at follow-up traits stratified according to children’s LTL tertiles. Supplementary table 4. Regression result for Cord blood LTL association with newborn and maternal glycaemic traits. Supplementary table 5. Sex-stratified maternal and offspring characteristics. Supplementary table 6. Glucose and insulin traits stratified according to LTL change group. Supplementary table 7. Sex -stratified children’s LTL associations with maternal and newborn characteristics. Supplementary table 8. Sex -stratified cord blood LTL associations with maternal and newborn characteristics. Supplementary table 9. Baseline characteristics between mothers returned for follow-up to mothers without follow-up. Supplementary figure 1. Diagram for HAPO and HAPO follow-up recruitment and mother-child pair excluded with reasons. Supplementary figure 2. The Heatmap showing the correlations between maternal (MA) and offspring (OS) glucose and insulin traits. Supplementary figure 3. Telomere length percentage change histogram. Supplementary figure 4. Schematic illustration on the conceptual associations between glucose and LTL. Supplementary figure 5. Correlations between maternal (MA) and offspring (OS) glucose levels. Supplementary figure 6. The calculation of glucose area under the curve. [file 12916_2022_2687_MOESM2_ESM.docx]

## Online-only supplementary file

## Supplementary Methods

Exclusion criteria for the HAPO study

The original multi-centre HAPO study had the following exclusion criteria 1. Aged less than 18 years old; 2. Planning to deliver in another hospital; 3. No ultrasonographic estimation within 6 to 24 gestational age and with an uncertain date to her last menstrual period; 5. Having more than one fetus; 6. Conception by using In vitro fertilization or gonadotropin ovulation; 7. Has been diagnosed diabetes during current pregnancy or performed glucose testing prior to the recruitment; 8. Has been diagnosed diabetes before current pregnancy and required medication treatment; 9. Enrolled into any other study that could interfere with the HAPO study; 10. Known infection with hepatitis B or C virus or human immunodeficiency virus (HIV); 11. Unable to converse in the languages used in the field centre without any help from an interpreter; 12. Previous participation in the HAPO study. For mothers with the following conditions, the OGTT results would have been unblinded: 1) 2-hr PG level diagnosed with DM which exceed 11.1 mmol/L, 2) Fasting PG level higher than 5.8 mmol/L. 3) Random PG level higher or equal to 8.9 mmol/L at 34-37 gestational weeks. 4) Or any PG level with less than 2.5 mmol/L. If there was any glucose measurement preformed outside the HAPO study setting after initial enrolment, participation would be terminated and their data were not included in the HAPO study.

Questionnaires and other background

At the prenatal visit, questions relating to parity, family history among first degree relatives (yes or no) and level of education (in form of year of education), alcohol consumption (yes or no) and smoking during pregnancy (yes or no), paternal height and weight were also collected. At follow up, parents of the children were asked to complete a questionnaire relating to perinatal history (e.g. breastfeeding history and duration), habitual dietary intake(frequency on consumption of fast food and sugar-sweetened beverages), sedentary lifestyle(habits on television, the Internet, video games and vigorous exercise) and frequency of physical activity (three categories: no exercise, regular exercise and frequent exercise).

Calculation for glucose and insulin and pancreatic response indices

1. Homeostasis model assessment of insulin resistance (HOMA-IR) was calculated as (FPI × FPG) / 22.5
2. Homeostasis model assessment of beta-cell function (HOMA-B) was calculated as FPI × 20 / (FPG - 3.5)
3. Insulinogenic index (IGI) was assessed as (insulin during OGTT at 30 min - 0 min) / (glucose during OGTT at 30 min - 0 min).
4. Matsuda index/ Insulin sensitivity index (ISI) was estimated using the formula proposed by Matsuda and DeFronzo (1): 10,000 / {square root of [FPG × FPI × (mean glucose during OGTT) × (mean insulin during OGTT)]}
5. Oral disposition index (DI) was calculated as (insulin during OGTT at 30 min - 0 min) / (glucose during OGTT at 30 min - 0 min) × (1 / HOMA-IR)
6. Beta-Cell function (BCF) was calculated with the equation IAUC/ GAUC; Areas under the curve for glucose (GAUC) and insulin (IAUC) during OGTT at 0-120 min were calculated using the trapezoid rule as shown.

The indices of HOMA-IR, HOMA-B, Glucose AUC at 0-120 min, Insulin AUC at 0-120 min, IGI, ISI and DI were the standard way to measure the β cell function (insulin response) and insulin sensitivity (insulin action).

GAUC calculation follows the method (Supplementary figure 6):

GAUC =Area A + Area B + Area C + Area D

= (glucose at 0 min + glucose at 15 min) × 15 / 2 + (glucose at 15 min + glucose at 30 min) × 15 / 2 + (glucose at 30 min + glucose at 60 min) × 30 / 2 + (glucose at 60 min + glucose at 120 min) × 60 / 2

= [(glucose at 0 min + glucose at 15 min × 2 + glucose at 30 min) × 15 + (glucose at 30 min + glucose at 60 min) × 30 + (glucose at 60 min + glucose at 120 min) × 60] / 2

A similar approach was done with the IAUC

One of the concerns might be the reliability of sensitivity index and pancreatic beta cell function among children. Previous research however had demonstrated high correlation between euglycemic clamp, a gold standard for assessing insulin sensitivity with these indices (2) (3) (4).

DNA quantitation and dilution

Nanodrop-2000 (ThermoFisher Scientific, Waltham, MA, USA) measurement was done before the DNA sample was used for analysis as well as the dilution of DNA. The process of real time-qPCR LTL measurement in brief: DNA was first diluted to 25ng/μl as the working concentration using nanodrop. Forward and reverse primers for both human beta globin gene (HBG) and telomere were ordered from Integrated DNA Technologies (Singapore). After adding all wells with 9μl master mix solution, 30 samples, negative control (NTC) with nuclease-free water and reference DNA (QC) were added in triplicate manner. All samples addition were done on ice. We have also put samples with both baseline cord DNA and 7-year-old child DNA onto the same plate to minimise the measurement variations. The 96-well plate would then be sealed, centrifuged and placed into an Applied Biosystems ViiA™ 7 Real‐Time PCR System (Life Technologies Corporation, Carlsbad, CA). Both reactions involve 2 stages: 1. 3min (95°C) 2. 15 seconds at 95°C and 1min annealing at 56°C (telomere) or 58°C (HBG), repeating for 40 cycles. The qPCR method was thoroughly optimised before the study. A steady decrease of the Ct values would be observed if increased amount of DNA was used.

Studies reported DNA extraction methods may have an influence on the LTL measurement. The LTL value for the same sample was reported longer, if it was extracted with the traditional phenol-chloroform method than with the column-based extraction method. Reassuringly, one study has reported no significant difference in TL between the two methods we used to extract DNA (5). It was also noted that maternal DNA was observed in umbilical cord blood. The amount however from a study using similarly real-time qPCR technology, has suggested that the maternal DNA fractional concentration in cord plasma was 3 x 10^-3^ (interquartile range, 1 x 10^-3^ to 1.6 x 10^-2^). This suggested the majority of the cord blood DNA is originated from the fetal DNA (6).

Quality Control

Coefficient of Variation (CV) for ΔΔCt, was calculated with the formula: CV = (Standard Deviation (SD) / Mean) × 100. Both NTC and QC reference DNA were used for adjusting inter-plate variability. Plates with more than ±1 cycle on telomere and HBG gene on QC would be rejected. The QC DNA was extracted from a male with hemochromatosis blood aged around 40-year-old undergoing one episode of therapeutic venesection. Multiple QC aliquots from one single blood sample were prepared and store at -80°C. For each testing samples, CVs were calculated in both telomere and HBG. If CV was higher than 2.5% for the triplicates, the sample result was first recalculated with the closest two results if less than 0.5 cycles difference between them. If CV remains higher than 2.5% for the closest two, both plates’ results for the particular samples would be rejected and the sample would be retested later. Thus, only samples test results with triplicates in both HBG and telomere plates less than 2.5% CV or duplicates with results less than 0.5 Ct values apart and CV less than 2.5% CV would be included in the data analysis. As such, we have repeated 41 DNA samples (3.26% of all the samples).

## Supplementary Results

Comparison between maternal baseline characteristics come back for follow-up

From the initial 1600 mothers, 970 subjects returned for follow-up. The mothers who came back for follow-up were older (p<0.001), with higher pre-pregnancy BMI (p=0.001), having higher pregnancy weight gain (p=0.023), and having a higher HbA1c (p=0.011), GAUC (p=0.021), OGTT glucose level at 120 minutes (p<0.001) (Supplementary table 8), when compared to those who did not return for follow-up for children at age 7.

Baseline characteristics with cord blood LTL

There were only insignificant positive associations observed between cord blood LTL and newborn anthropometric indices, with ponderal index (β=0.032±0.018, p=0.068) (Supplementary table 1). Such associations persisted after adjusting for offspring sex and age. No association however was observed with birthweight (β=0.000114±0.0001, p=0.265). Insignificant associations between longer cord blood LTL and increased parity (β=0.107±0.062, p=0.083), as well as C-section delivered offspring (β=0.171±0.095, p=0.074), were also observed.

Baseline characteristics included for confounders

Apart from the aforementioned confounders: offspring sex, age, exercise level and maternal age at conception which have shown association with telomere length, other confounders including maternal pre-pregnant BMI, maternal GAUC during pregnancy, parity, current maternal hypertensive status for blood pressure, current maternal and paternal diabetes status for glucose, insulin levels and pancreatic function traits, C-section or not, gestational age at delivery, history of breast feeding, birthweight, childhood BMI were included into model 2 or model 3 of the regression model for adjustments. The inclusion of the confounders was based on the objective to adjust for the associations between offspring LTL and the independent variables, which were reported, or could potentially affect the association. In model 1, offspring sex and age were adjusted as a basic confounder adjustment between LTL and traits. For model 2, perinatal exposures such as maternal age at conception, maternal pre-pregnant BMI (7), maternal AUCglu during pregnancy, parity (8) and gestational age at delivery were included. Current maternal hypertensive status for blood pressure or current maternal and paternal diabetes status were adjusted specifically for blood pressure (9) or OGTT glucose, insulin levels and pancreatic function traits as these traits were reported directly affected by their mothers to the offspring. On top of the perinatal exposure, post-natal exposures such as C-section delivery, history of breastfeeding (10), birthweight, childhood BMI (11) were also included, based on reported associations.

| Supplementary table 1 Univariate associations between cord and children’s LTL with their basic characteristics | | | | | | |  |  |
| --- | --- | --- | --- | --- | --- | --- | --- | --- |
|  | **Cord blood LTL (n=313)** | | | **7-year-old LTL (n=882)** | | | | |
|  | **Unstd Beta** | **SE** | **P** | **Unstd Beta** | **SE** | **P** | |  |
| Maternal age EDC | 0.008 | 0.009 | 0.333 | 0.013 | 0.003 | **<0.001** | |  |
| Maternal pre-pregnancy BMI | -0.005 | 0.013 | 0.703 | 0.001 | 0.006 | 0.822 | |  |
| Maternal SBP | -0.005 | 0.004 | 0.211 | -0.001 | 0.002 | 0.649 | |  |
| Maternal GAUC | 0.022 | 0.018 | 0.221 | 0.021 | 0.008 | **0.007** | |  |
| Ponderal index | 0.032 | 0.018 | 0.068 | -0.007 | 0.007 | 0.366 | |  |
| C-section (yes/no) | 0.171 | 0.095 | 0.074 | 0.062 | 0.039 | 0.109 | |  |
| Gestational age at delivery | 0.007 | 0.033 | 0.827 | -0.005 | 0.014 | 0.718 | |  |
| Parity | 0.107 | 0.062 | 0.083 | 0.035 | 0.026 | 0.17 | |  |
| Offspring sex (female = 1) | 0.037 | 0.074 | 0.62 | 0.116 | 0.032 | **<0.001** | |  |
| Follow up age | N.A. | N.A. | N.A. | -0.047 | 0.037 | 0.2 | |  |
| Childhood Exercise level  (0,1, 2 with 0 = lowest) | N.A. | N.A. | N.A. | -0.022 | 0.007 | **<0.001** | |  |
| Childhood BMI at 7 | N.A. | N.A. | N.A. | -0.016 | 0.007 | **0.02** | |  |
| Childhood SBP at 7 | N.A. | N.A. | N.A. | -0.0003 | 0.002 | 0.891 | |  |
| Maternal DM status at FU | 0.244 | 0.357 | 0.495 | 0.04 | 0.138 | 0.771 | |  |
| Paternal DM status at FU | 0.103 | 0.112 | 0.355 | 0.004 | 0.052 | 0.942 | |  |

Supplementary table 2 Offspring LTL at follow up and associations with glucose and insulin related traits

|  | **Model 0** | | | **Model 1** | | **Model 2** | | **Model 3** | |
| --- | --- | --- | --- | --- | --- | --- | --- | --- | --- |
|  | **Beta** | **SE** | **P** | **Beta** | **P** | **Beta** | **P** | **Beta** | **P** |
| Fasting glucose (mmol/l) | -0.065 | 0.045 | 0.146 | -0.012 | 0.474 | -0.015 | 0.363 | -0.017 | 0.326 |
| 1 h glucose (mmol/l) | 0.028 | 0.011 | **0.009** | 0.041 | 0.014 | 0.037 | 0.03 | 0.038 | 0.025 |
| 2 h glucose (mmol/l) | 0.06 | 0.017 | **0.001** | 0.054 | **0.001** | 0.058 | **0.001** | 0.06 | **<0.001** |
| AUCglu at 0–120 min | 0.00044 | 0.00014 | **0.002** | 0.051 | **0.002** | 0.048 | **0.005** | 0.05 | **0.003** |
| Fasting Insulin (mIU/L) | -0.002 | 0.003 | 0.606 | -0.03 | 0.072 | -0.018 | 0.287 | -0.023 | 0.166 |
| 1 h insulin (mIU/L) | -0.001 | 0.001 | 0.173 | -0.038 | 0.022 | -0.03 | 0.082 | -0.03 | 0.079 |
| 2 h insulin (mIU/L) | -0.001 | 0.001 | 0.272 | -0.027 | 0.104 | -0.016 | 0.348 | -0.004 | 0.821 |
| AUCins at 0–120 min | -1.2E-05 | 0.000007 | 0.081 | -0.033 | 0.05 | -0.025 | 0.154 | -0.02 | 0.235 |
| Fasting C-pep (ug/L) | -0.126 | 0.04 | **0.002** | -0.054 | **0.001** | -0.037 | 0.028 | -0.031 | 0.072 |
| HOMA-BCF | -0.001 | 0.00023 | 0.025 | -0.042 | **0.011** | -0.03 | 0.075 | -0.027 | 0.114 |
| Insulinogenic index 30 | -0.0005 | 0.00018 | **0.007** | -0.038 | 0.024 | -0.032 | 0.066 | -0.029 | 0.086 |
| Disposition Index | -0.001 | 0.002 | 0.472 | -0.016 | 0.352 | -0.017 | 0.326 | -0.018 | 0.295 |
| Beta-Cell function | -0.002 | 0.001 | **0.007** | -0.042 | **0.011** | -0.034 | 0.048 | -0.029 | 0.09 |
| HOMA-IR | -0.065 | 0.021 | **0.002** | -0.046 | **0.005** | -0.033 | 0.047 | -0.038 | 0.026 |
| Matsuda Index | 0.004 | 0.002 | 0.042 | 0.038 | 0.022 | 0.031 | 0.074 | 0.018 | 0.289 |

Model 0: unadjusted; Model 1 (basic): adjusted for sex and age at childhood Model 2 (parental effects): Model 1 + maternal prepregnant BMI, maternal age at EDC, maternal AUCglu during pregnancy, parity; for glucose and insulin levels, as well as indices for pancreatic function, they were further adjusted for current maternal and paternal diabetes status. Model 3 (perinatal and childhood environmental factors): Model 2 + C-section or not, gestational age at delivery, history of breast feeding, birthweight, childhood BMI and childhood exercise level (0, 1, 2) The values in model 1 and 2 subjected to normal inverse transformation, their SE were all 0.016-0.017.

Supplementary table 3 Basic characteristics offspring at follow-up traits stratified according to children’s LTL tertiles

|  | **Tertile 1 (n=294)** | **Tertile 2 (n=294)** | **Tertile 3 (n=294)** | | **Total (n=882)** | |  | | |
| --- | --- | --- | --- | --- | --- | --- | --- | --- | --- |
| Children’s LTL (T/S ratio) | 0.96 ± 0.32 | 1.50 ± 0.11 | 2.00 ± 0.26 | 1.48 ± 0.49 | |  | | |  |
| Fasting Glucose (mmol/l) | 4.6 ± 0.3 | 4.6 ± 0.4 | 4.5 ± 0.4 | 4.6 ± 0.4 | | 0.11 | | |  |
| 2-hour glucose (mmol/l) | 5.2 ± 1.0 | 5.4 ± 0.9 | 5.4 ± 1.0 | 5.3 ± 1.0 | | **0.009** | | |  |
| AUCglu at 0–120 min | 719.8 ± 114.2 | 747.4 ± 115.8 | 745.1 ± 122.8 | 737.3 ± 118.2 | | **0.01** | | |  |
| Fasting Insulin (mIU/L)* | 2.3[2.0-4.8] | 2.2[2.0-5.1] | 2.0[2.0-3.7] | 2.0[2.0-4.5] | | **0.012** | | |  |
| 2-hour insulin (mIU/L)* | 17.1[9.7-28.1] | 18.5[10.2-29.3] | 15.2[9.1-24.1] | 16.9[9.8-27.6] | | 0.191 | | |  |
| AUCins at 0–120 min* | 2463.9[1629.9-3740.1] | 2384.3[1471.8-3890.8] | 2143.1[1473.5-3324.1] | 2307.3[1513.5-3652.3] | | 0.027 | | |  |
| Fasting C-peptide (ug/l)* | 0.2[0.1-0.6] | 0.2[0.1-0.5] | 0.1[0.1-0.4] | 0.2[0.1-0.5] | | **0.002** | | |  |
| HOMA-BCF* | 53.8[39.3-96.4] | 57.1[40.0-86.5] | 50.0[40.0-80.0] | 52.33[40.0-86.1] | | 0.13 | | |  |
| Insulinogenic index* | 65.71[32.5-114.6] | 50.5[25.8-96.5] | 46.7[22.3-90.7] | 54.16[25.4-101.0] | | **0.006** | | |  |
| Disposition Index* | 6.1[3.6-10.3] | 5.5[3.1-9.0] | 5.6[2.8-9.3] | 5.8[3.2-9.4] | | 0.106 | | |  |
| Beta-Cell function* | 23.7[16.2-38.0] | 22.7[14.0-35.9] | 20.2[14.5-30.1] | 22.4[14.7-34.7] | | **0.005** | | |  |
| HOMA-IR* | 0.5[0.4-1.0] | 0.5[0.4-1.0] | 0.4[0.4-0.8] | 0.4[0.4-0.9] | | **0.003** | | |  |
| Matsuda Index* | 14.4[9.2-20.1] | 13.9[8.7-20.4] | 16.4[10.9-21.6] | 15.0[9.4-20.5] | | 0.016 | | |  |
| *shown with median [Q1-Q3] PLUS p-values comparison with natural log transformation in correlation test  P shown at the last column refers to the P value for trend between T1, T2 and T3 only. | | | | | | | |  |  |

Supplementary table 4 Regression result for Cord blood LTL association with newborn and maternal glycaemic traits

|  | **Model 0** | | | **Model 1** | | | **Model 2** | | | **Model 3** | | |
| --- | --- | --- | --- | --- | --- | --- | --- | --- | --- | --- | --- | --- |
|  | **Beta** | **SE** | **P** | **Beta** | **SE** | **P** | **Beta** | **SE** | **P** | **Beta** | **SE** | **P** |
| Maternal HbA1c at OGTT | -0.079 | 0.096 | 0.411 | -0.03 | 0.035 | 0.389 | -0.043 | 0.035 | 0.224 | -0.047 | 0.035 | 0.184 |
| Maternal fasting glucose | -0.219 | 0.103 | **0.034** | -0.072 | 0.035 | **0.038** | -0.087 | 0.034 | **0.011** | -0.087 | 0.034 | **0.01** |
| Maternal glucose 60 min | 0.008 | 0.022 | 0.723 | 0.019 | 0.036 | 0.6 | 0.007 | 0.036 | 0.838 | 0.003 | 0.036 | 0.923 |
| Maternal glucose 120 min | 0.021 | 0.027 | 0.455 | 0.026 | 0.037 | 0.484 | 0.018 | 0.036 | 0.615 | 0.019 | 0.037 | 0.611 |
| Maternal OGTT GAUC | 0.005 | 0.017 | 0.745 | 0.018 | 0.037 | 0.619 | 0.006 | 0.036 | 0.88 | 0.002 | 0.036 | 0.956 |
| Maternal GDM IADPSG (%) | 0.005 | 0.098 | 0.958 | -0.003 | 0.035 | 0.943 | -0.009 | 0.035 | 0.796 | -0.007 | 0.035 | 0.842 |

Model 0: unadjusted; Model 1 (basic): adjusted for sex and gestational age; Model 2 (newborn characteristics): Model 1 + birthweight and C-section delivery or not; Model 3 (parental effects): Model 2 + maternal pre-pregnacy BMI, parity, current maternal and paternal diabetes or not.

| Supplementary table 5 Sex-stratified maternal and offspring characteristics | | | | |
| --- | --- | --- | --- | --- |
| **Maternal characteristics** | **Male (n=461)** | **Female (n=421)** | **p** |  |
| Children’s Telomere length  (Delta delta Ct) | 1.42 ± 0.50 | 1.55 ± 0.48 | **0.001** |  |
| Maternal age EDC (year) | 31.4 ± 4.7 | 31.2 ± 4.6 | 0.683 |  |
| Pre-pregnant BMI (kg/m^2^) | 20.9 ± 2.7 | 20.9 ± 3.0 | 0.868 |  |
| Prenatal smoking# | 8 (1.7) | 5 (1.2) | 0.501 |  |
| Parity, 0 vs ≥ 1, 0# | 272 (59.0) | 244 (58.0) | 0.880 |  |
| Maternal fasting glucose | 4.4 ± 0.3 | 4.4 ± 0.3 | 0.539 |  |
| Maternal DM at FU# | 42 (9.1) | 56 (13.3) | 0.048 |  |
| Paternal DM at FU# | 47 (10.2) | 47 (11.2) | 0.640 |  |
| C-section# | 123 (26.7) | 88 (20.9) | 0.045 |  |
| Breastfeeding# | 228 (49.5) | 210 (49.9) | 0.870 |  |
| Exercise level (0, 1, 2) | 2.3 ± 0.6 | 2.2 ± 0.6 | 0.111 |  |
| Maternal OGTT GAUC | 13.1 ± 2.1 | 13.2 ± 2.3 | 0.456 |  |
| **Children characteristics** | **Male** | **Female** | **p** |  |
| Follow-up age (year) | 6.9 ± 0.5 | 7.0 ± 0.4 | 0.098 |  |
| BMI (kg/m2) | 15.4 ± 2.4 | 14.8 ± 2.1 | **<0.001** |  |
| Fasting Glucose (mmol/l) | 4.7 ± 0.4 | 4.5 ± 0.4 | **<0.001** |  |
| 2-hour glucose (mmol/l) | 5.3 ± 1.0 | 5.3 ± 1.0 | 0.357 |  |
| AUCglu at 0–120 min | 735.7 ± 117.8 | 739.3 ± 120.8 | 0.663 |  |
| Fasting Insulin (mIU/L)* | 2.1 [2.0, 4.7] | 2.0 [2.0, 4.2] | 0.412 |  |
| 2-hour insulin (mIU/L)* | 16.3 [9.5, 25.9] | 17.1 [10.1, 29.8] | 0.054 |  |
| AUCins at 0–120 min* | 2,293.0 [1,518.5, 3,626.7] | 2,387.6 [1,511.0, 3,653.6] | 0.824 |  |
| HOMA-BCF* | 50.0 [36.4, 81.9] | 57.1 [43.4, 89.1] | **<0.001** |  |
| Insulinogenic index* | 58.3 [25.8, 112.8] | 48.7 [25.3, 92.8] | 0.114 |  |
| Disposition Index* | 6.1 [3.2, 10.0] | 5.5 [3.1, 8.7] | 0.056 |  |
| Beta-Cell function* | 22.5 [15.2, 34.3] | 22.3 [14.5, 36.1] | 0.927 |  |
| HOMA-IR* | 0.5 [0.4, 1.0] | 0.4 [0.4, 0.9] | **0.002** |  |
| Matsuda Index* | 14.5 [9.3, 20.5] | 15.3 [9.7, 20.6] | 0.371 |  |

*shown median [Q1-Q3] PLUS p-values comparison with natural log transformation in correlation test

#shown in number (percentage)

Supplementary table 6 Glucose and insulin traits stratified according to LTL change group

| **Offspring characteristics at 7** | |  |  |  |
| --- | --- | --- | --- | --- |
| **Lengthened(n=50)** | | **Maintained (n=148)** | **Shortened (n=110)** | **P** |
| Cord blood LTL (kbp) | 16.5 ± 3.3 | 22.0 ± 3.4 | 23.3 ± 4.1 | <0.001 |
| Children's LTL (kbp) | 21.4 ± 3.0 | 21.6 ± 3.2 | 18.2 ± 3.4 | <0.001 |
| LTL percentage change | -23.9 ± 16.6 | 1.3 ± 4.4 | 17.2 ± 7.3 | <0.001 |
| Offspring FBG (mmol/l) | 4.6 ± 0.4 | 4.6 ± 0.3 | 4.7 ± 0.4 | **0.042** |
| 1-hour glucose (mmol/l) | 6.5 ± 1.5 | 5.9 ± 1.5 | 5.7 ± 1.5 | **0.024** |
| 2-hour glucose (mmol/l) | 5.5 ± 0.9 | 5.4 ± 0.9 | 5.2 ± 1.0 | 0.256 |
| AUCglu at 0–120 min | 773.0 ± 113.7 | 739.3 ± 107.7 | 729.9 ± 108.1 | 0.072 |
| Fasting Insulin (mIU/L)* | 2.0 [2.0, 2.3] | 2.0 [2.0, 3.6] | 2.7 [2.0, 4.9] | **0.002** |
| 1-hour insulin (mIU/L)* | 16.1 [12.6, 28.4] | 18.7 [11.1, 28.9] | 20.9 [12.3, 35.1] | 0.212 |
| 2-hour insulin (mIU/L)* | 13.7 [9.9, 20.2] | 17.2 [11.1, 26.8] | 17.9 [10.7, 30.1] | 0.165 |
| AUCins at 0–120 min* | 2,128.1 [1,476.2, 2,765.7] | 2,379.4 [1,542.2, 3,190.9] | 2,720.4 [1,868.3, 3,830.5] | **0.009** |
| HOMA-BCF* | 47.2 [33.3, 75.4] | 50.0 [40.0, 71.5] | 52.3 [36.4, 88.5] | 0.507 |
| Insulinogenic index* | 38.8 [12.6, 60.1] | 47.8 [26.5, 92.1] | 63.7 [33.7, 108.2] | **0.001** |
| Disposition Index* | 4.5 [2.1, 7.9] | 6.0 [3.4, 9.9] | 5.7 [3.6, 9.0] | **0.027** |
| Beta-Cell function* | 19.4 [13.8, 24.9] | 21.8 [15.2, 30.3] | 25.3 [18.1, 38.7] | **0.002** |
| HOMA-IR* | 0.4 [0.4, 0.5] | 0.4 [0.4, 0.8] | 0.5 [0.4, 1.0] | **0.001** |
| Matsuda Index* | 17.8 [13.5, 21.0] | 15.4 [11.3, 20.0] | 13.3 [9.1, 18.0] | **0.01** |
| **Maternal characteristics during pregnancy** | |  |  |  |
| Maternal HbA1c at OGTT | 5.0 ± 0.4 | 4.9 ± 0.4 | 4.9 ± 0.3 | 0.246 |
| Maternal FBG (mmol/L) | 4.5 ± 0.4 | 4.4 ± 0.3 | 4.4 ± 0.3 | 0.23 |
| 1-hr Maternal glucose (mmol/l) | 8.0 ± 1.8 | 7.6 ± 1.5 | 7.6 ± 1.5 | 0.368 |
| 2-hr Maternal glucose (mmol/l) | 6.9 ± 1.3 | 6.6 ± 1.3 | 6.7 ± 1.3 | 0.577 |
| Maternal OGTT GAUC | 13.6 ± 2.2 | 13.1 ± 2.1 | 13.2 ± 1.9 | 0.322 |
| Maternal GDM IADPSG (%) | 12 (24.0) | 18 (12.2) | 15 (13.6) | 0.116 |

* Reported in form of median [Q1, Q3] as the trait is not normally distribute.

Supplementary table 7 Sex -stratified children’s LTL associations with maternal and newborn characteristics

|  | | **Model 0** | | | | | | | | | | | **Model 1** | | | | | | | | **Model 2** | | | | | | | | **Model 3** | | | | | | | | |  |  | |  | Model 2 |
| --- | --- | --- | --- | --- | --- | --- | --- | --- | --- | --- | --- | --- | --- | --- | --- | --- | --- | --- | --- | --- | --- | --- | --- | --- | --- | --- | --- | --- | --- | --- | --- | --- | --- | --- | --- | --- | --- | --- | --- | --- | --- | --- |
|  | | **Male** | | | | | | **Female** | | | | | **Male** | | | | **Female** | | | | **Male** | | | **Female** | | | | | **Male** | | **Female** | | | | | | |  | |  |  |  |
|  | | **Beta** | | **SE** | | **P** | | **Beta** | | **SE** | | **P** | **Beta** | | **P** | | | **Beta** | | **P** | **Beta** | | **P** | | **Beta** | | **P** | | **Beta** | **P** | | **Beta** | | **P** | | |  |  |  |  |  |  |
| Maternal HbA1c at OGTT | -0.012 | | 0.066 | | 0.858 | | -0.105 | | 0.069 | | 0.129 | | -0.018 | 0.861 | | -0.156 | | | 0.157 | | -0.042 | 0.676 | | | | -0.115 | | 0.296 | -0.115 | 0.26 | | | -0.15 | | 0.176 |  |  |  |  |  |  |  |
| Maternal fasting glucose | 0.135 | | 0.071 | | 0.059 | | 0.14 | | 0.069 | | **0.042** | | 0.164 | 0.092 | | 0.223 | | | **0.032** | | 0.134 | 0.173 | | | | 0.243 | | **0.019** | 0.093 | 0.345 | | | 0.218 | | **0.039** |  |  |  |  |  |  |  |
| Maternal glucose 60 min | 0.022 | | 0.015 | | 0.141 | | 0.02 | | 0.014 | | 0.158 | | 0.142 | 0.14 | | 0.126 | | | 0.234 | | 0.128 | 0.187 | | | | 0.121 | | 0.252 | 0.072 | 0.471 | | | 0.08 | | 0.445 |  |  |  |  |  |  |  |
| Maternal glucose 120 min | 0.022 | | 0.019 | | 0.237 | | 0.045 | | 0.016 | | **0.006** | | 0.062 | 0.502 | | 0.293 | | | **0.008** | | 0.053 | 0.563 | | | | 0.287 | | **0.01** | 0.017 | 0.86 | | | 0.232 | | **0.034** |  |  |  |  |  |  |  |
| Maternal OGTT GAUC | 0.018 | | 0.011 | | 0.105 | | 0.021 | | 0.01 | | **0.041** | | 0.132 | 0.16 | | 0.186 | | | 0.085 | | 0.12 | 0.206 | | | | 0.183 | | 0.091 | 0.071 | 0.476 | | | 0.14 | | 0.184 |  |  |  |  |  |  |  |
| Maternal GDM_IADPSG (%) | 0.061 | | 0.072 | | 0.397 | | 0.096 | | 0.061 | | 0.116 | | -0.063 | 0.366 | | 0.086 | | | 0.427 | | -0.034 | 0.675 | | | | 0.086 | | 0.42 | -0.11 | 0.241 | | | 0.039 | | 0.72 |  |  |  |  |  |  |  |

Model 0: unadjusted; Model 1 (basic): adjusted for age at childhood;

Model 2 (perinatal and childhood environmental factors): Model 1 + C-section or not, history of breast feeding, childhood BMI and childhood exercise level (0, 1, 2); Model 3 (parental and newborn effects): Model 2 + maternal prepregnant BMI, maternal age at EDC, parity, birthweight and current mother and father diabetes or not. The values in model 1 and 2 subjected to normal inverse transformation, their SE were all 0.010-0.011 and were ignored from the table.

Supplementary table 8 Sex -stratified cord blood LTL associations with maternal and newborn characteristics

|  | **Model 0** | | | | | | **Model 1** | | | | | | **Model 2** | | | | | |  |  |
| --- | --- | --- | --- | --- | --- | --- | --- | --- | --- | --- | --- | --- | --- | --- | --- | --- | --- | --- | --- | --- |
|  | **Male** | | | **Female** | | | **Male** | | | **Female** | | | **Male** | | | **Female** | | | |  |
|  | **Beta** | **SE** | **P** | **Beta** | **SE** | **P** | **Beta** | **SE** | **P** | **Beta** | **SE** | **P** | **Beta** | **SE** | **P** | **Beta** | **SE** | **P** | | |
| Maternal HbA1c at OGTT | 0.169 | 0.159 | 0.289 | -0.329 | 0.156 | **0.037** | 0.037 | 0.049 | 0.45 | -0.104 | 0.05 | **0.039** | 0.003 | 0.05 | 0.948 | -0.098 | 0.05 | 0.051 | | |
| Maternal fasting glucose | -0.182 | 0.149 | 0.224 | -0.332 | 0.161 | **0.04** | -0.066 | 0.052 | 0.202 | -0.077 | 0.047 | 0.102 | -0.092 | 0.049 | 0.063 | -0.081 | 0.047 | 0.084 | | |
| Maternal glucose 60 min | 0.03 | 0.038 | 0.44 | 0.04 | 0.034 | 0.241 | -0.0002 | 0.054 | 0.997 | 0.034 | 0.049 | 0.49 | -0.029 | 0.052 | 0.577 | 0.035 | 0.05 | 0.484 | | |
| Maternal glucose 120 min | 0.065 | 0.049 | 0.188 | 0.034 | 0.04 | 0.403 | 0.025 | 0.057 | 0.666 | 0.026 | 0.048 | 0.593 | -0.001 | 0.057 | 0.985 | 0.032 | 0.049 | 0.507 | | |
| Maternal OGTT GAUC | 0.029 | 0.029 | 0.329 | 0.024 | 0.025 | 0.33 | 0.004 | 0.057 | 0.948 | 0.031 | 0.048 | 0.524 | -0.033 | 0.055 | 0.548 | 0.032 | 0.049 | 0.516 | | |
| Maternal GDM | 0.115 | 0.183 | 0.531 | 0.026 | 0.143 | 0.857 | 0.058 | 0.053 | 0.27 | -0.033 | 0.047 | 0.489 | -0.027 | 0.054 | 0.617 | 0.004 | 0.048 | 0.931 | | |

Model 0: unadjusted; Model 1 (basic): adjusted for gestational age; Model 2 (newborn): Model 1 + Ponderal index, and C-section delivery or not, maternal pre-pregnacy BMI, parity, current maternal and paternal diabetes

Supplementary table 9 Baseline characteristics between mothers returned for follow-up to mothers without follow-up

|  | **Without follow up (n=630)** | **With follow-up (n=970)** | **P** |
| --- | --- | --- | --- |
| Maternal age EDC (year) | 30.4 ± 5.2 | 31.3 ± 4.6 | **<0.001** |
| Prenatal smoking (%) | 19 (2.9) | 16 (1.6) | 0.12 |
| Maternal current DM (%) | 82 (12.3) | 117 (11.8) | 0.945 |
| Paternal current DM (%) | 56 (8.4) | 106 (10.7) | 0.255 |
| Parity, 0 vs ≥ 1, 0(%) | 419 (63.0) | 592 (59.6) | 0.4 |
| C-section (%) | 140 (21.1) | 193 (19.4) | 0.083 |
| Pre-pregnant BMI (kg/m^2^) | 20.4 ± 2.8 | 20.9 ± 2.9 | **0.001** |
| Maternal weight gain (kg) | 15.6 ± 4.9 | 15.1 ± 4.4 | **0.023** |
| Maternal HbA1c at OGTT | 4.9 ± 0.4 | 5.00 ± 0.4 | **0.011** |
| Maternal fasting glucose (mmol/L) | 4.4 ± 0.4 | 4.4 ± 0.4 | 0.507 |
| Maternal glucose 60 min (mmol/L) | 7.6 ± 1.8 | 7.72 ± 1.7 | 0.132 |
| Maternal glucose 120 min (mmol/L) | 6.4 ± 1.3 | 6.69 ± 1.36 | **<0.001** |
| Maternal OGTT GAUC (in hour) | 13.0± 2.4 | 13.3 ± 2.3 | **0.021** |
| Maternal GDM (IADPSG) = 1 (%) | 85 (12.8) | 155 (15.6) | 0.125 |
| Maternal SBP (mmhg) | 100.8 ± 9.7 | 101.4 ± 9.9 | 0.209 |
| Offspring sex (female %) | 293 (47.2) | 442 (48.1) | 0.815 |

Supplementary figure 1 Diagram for HAPO and HAPO follow-up recruitment and mother-child pair excluded with reasons


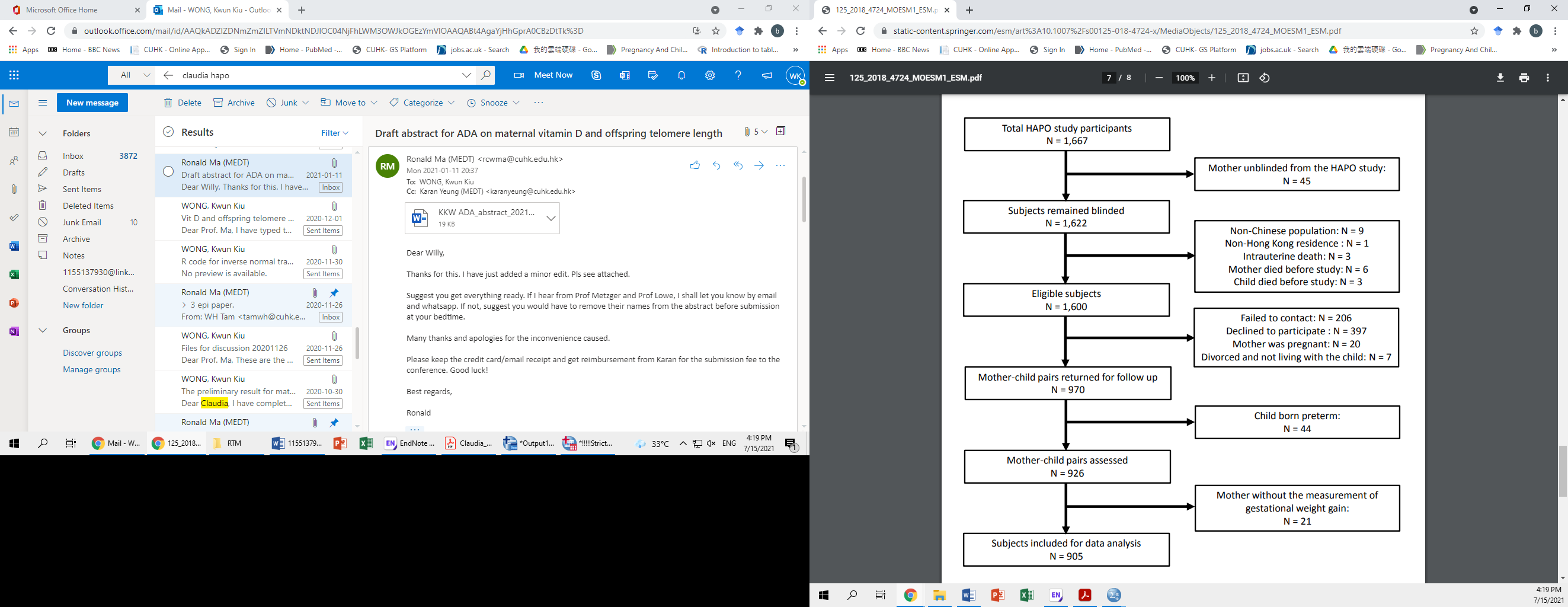


Supplementary figure 2 The Heatmap showing the correlations between maternal (MA) and offspring (OS) glucose and insulin traits


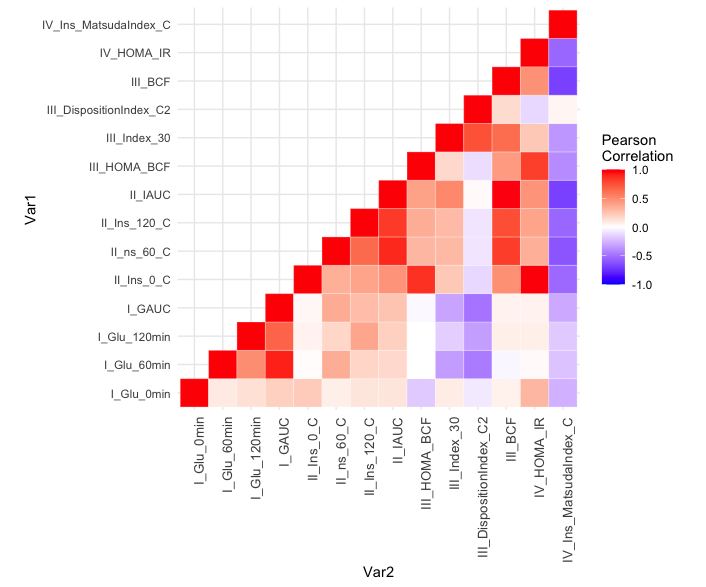


Supplementary figure 3 Telomere length percentage change histogram


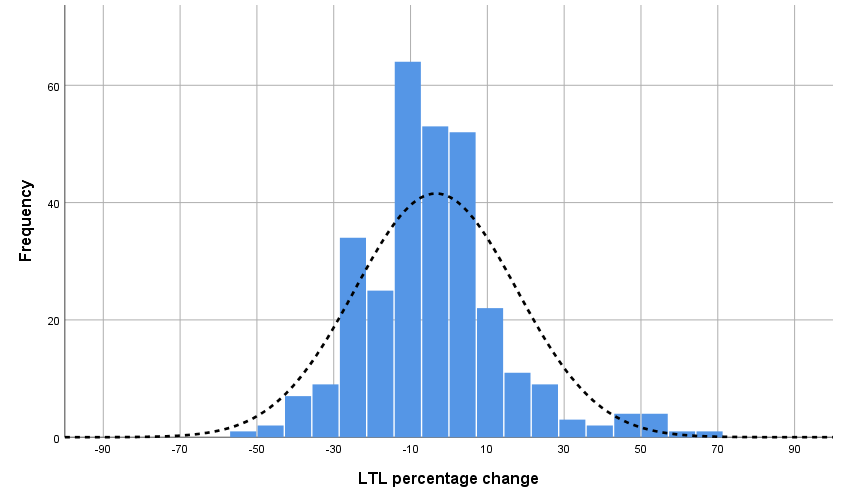


Supplementary figure 4 Schematic illustration on the conceptual associations between glucose and LTL


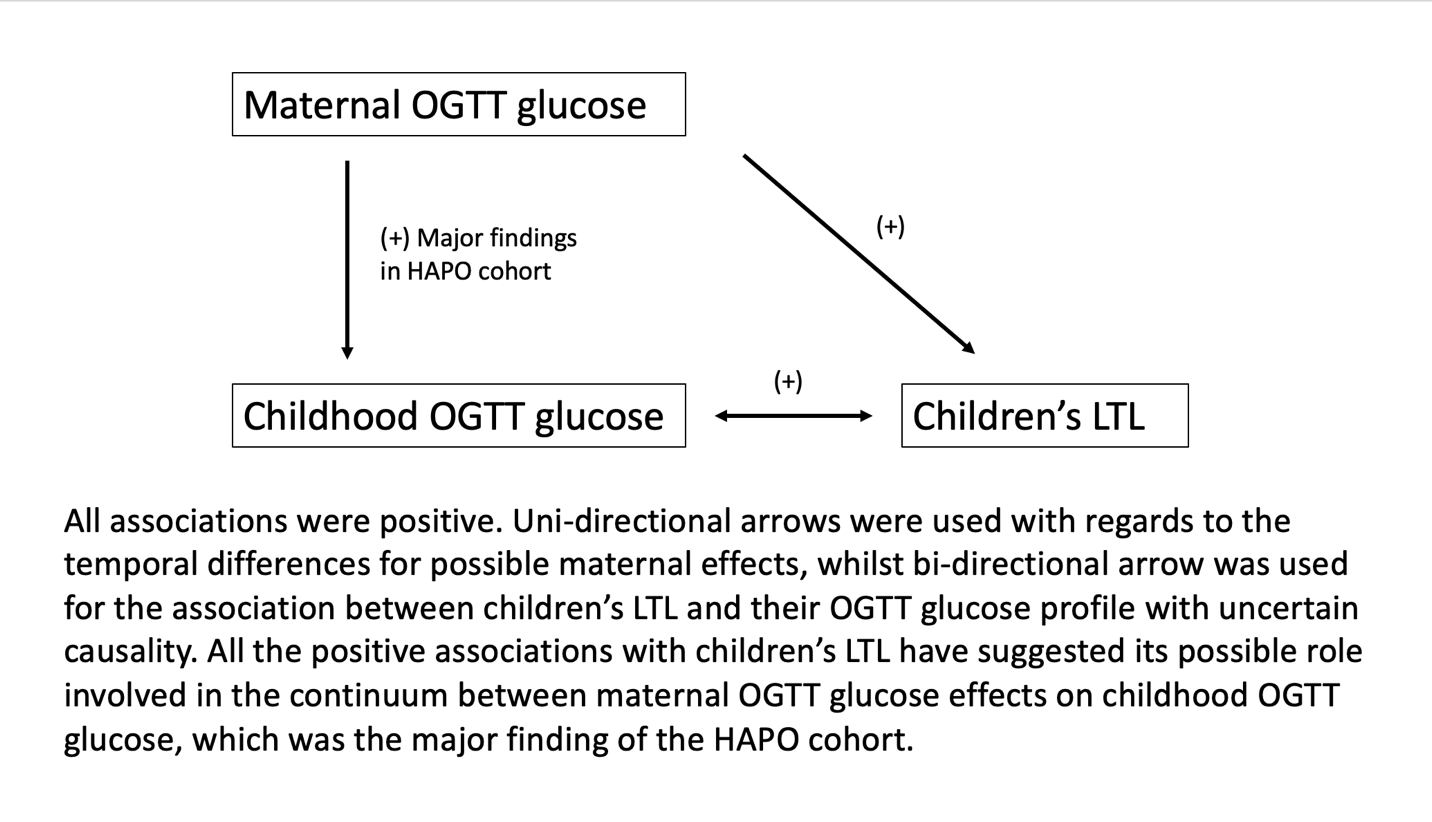


Supplementary figure 5 Correlations between maternal (MA) and offspring (OS) glucose levels


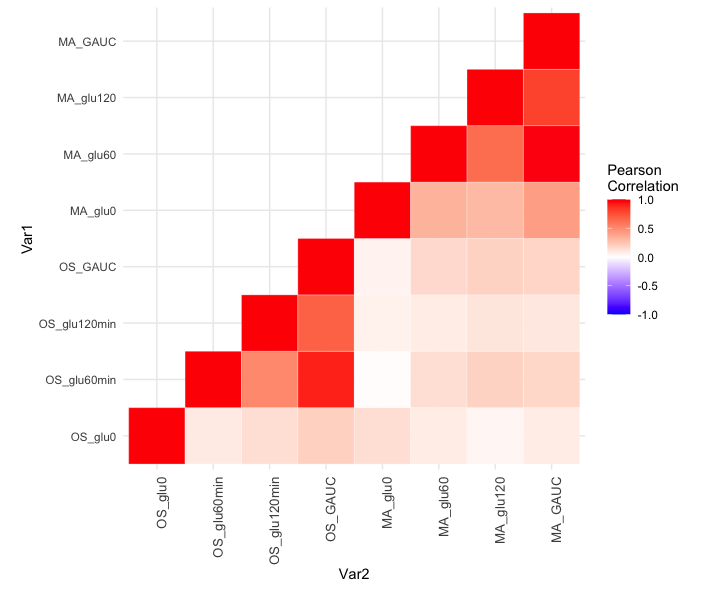


Supplementary figure 6 The calculation of glucose area under the curve
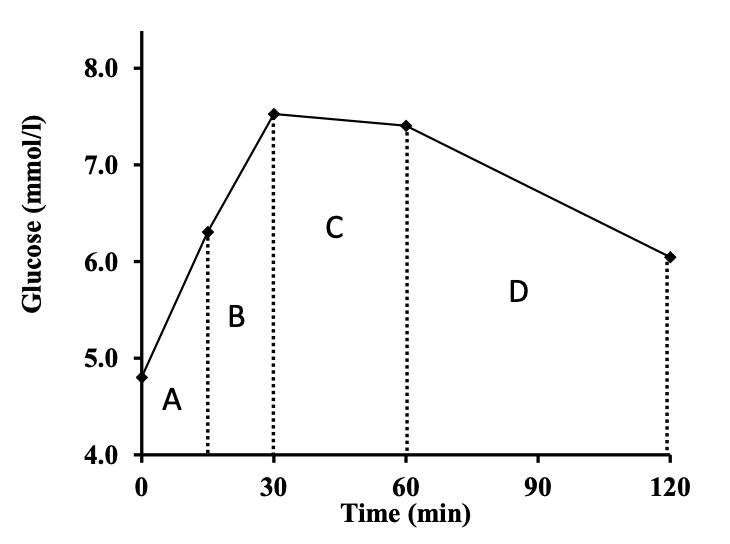


Reference

1. Gastaldelli A, Ferrannini E, Miyazaki Y, Matsuda M, DeFronzo RA, San Antonio metabolism s. Beta-cell dysfunction and glucose intolerance: results from the San Antonio metabolism (SAM) study. Diabetologia. 2004;47(1):31-9.

2. Gungor N, Saad R, Janosky J, Arslanian S. Validation of surrogate estimates of insulin sensitivity and insulin secretion in children and adolescents. The Journal of pediatrics. 2004;144(1):47-55.

3. Yeckel CW, Weiss R, Dziura J, Taksali SE, Dufour S, Burgert TS, et al. Validation of insulin sensitivity indices from oral glucose tolerance test parameters in obese children and adolescents. The Journal of Clinical Endocrinology & Metabolism. 2004;89(3):1096-101.

4. Uwaifo GI, Fallon EM, Chin J, Elberg J, Parikh SJ, Yanovski JA. Indices of insulin action, disposal, and secretion derived from fasting samples and clamps in normal glucose-tolerant black and white children. Diabetes care. 2002;25(11):2081-7.

5. Cunningham JM, Johnson RA, Litzelman K, Skinner HG, Seo S, Engelman CD, et al. Telomere length varies by DNA extraction method: implications for epidemiologic research. Cancer Epidemiology and Prevention Biomarkers. 2013;22(11):2047-54.

6. Lo YD, Lau TK, Chan LY, Leung TN, Chang AM. Quantitative analysis of the bidirectional fetomaternal transfer of nucleated cells and plasma DNA. Clinical chemistry. 2000;46(9):1301-9.

7. Martens DS, Plusquin M, Gyselaers W, De Vivo I, Nawrot TS. Maternal pre-pregnancy body mass index and newborn telomere length. BMC medicine. 2016;14(1):1-10.

8. Pollack A, Rivers K, Ahrens K. Parity associated with telomere length among US reproductive age women. Human Reproduction. 2018;33(4):736-44.

9. Miliku K, Bergen NE, Bakker H, Hofman A, Steegers EA, Gaillard R, et al. Associations of maternal and paternal blood pressure patterns and hypertensive disorders during pregnancy with childhood blood pressure. Journal of the American Heart Association. 2016;5(10):e003884.

10. Wojcicki JM, Heyman MB, Elwan D, Lin J, Blackburn E, Epel E. Early exclusive breastfeeding is associated with longer telomeres in Latino preschool children. The American Journal of Clinical Nutrition. 2016;104(2):397-405.

11. Clemente DB, Maitre L, Bustamante M, Chatzi L, Roumeliotaki T, Fossati S, et al. Obesity is associated with shorter telomeres in 8 year-old children. Scientific reports. 2019;9(1):1-8.

12. Aguiar SS, Sousa CV, Santos PA, Barbosa LP, Maciel LA, Coelho-Júnior HJ, et al. Master athletes have longer telomeres than age-matched non-athletes. A systematic review, meta-analysis and discussion of possible mechanisms. Experimental Gerontology. 2020:111212.

13. Shadyab AH, LaMonte MJ, Kooperberg C, Reiner AP, Carty CL, Manini TM, et al. Leisure-time physical activity and leukocyte telomere length among older women. Experimental gerontology. 2017;95:141-7.

14. Lamprokostopoulou A, Moschonis G, Manios Y, Critselis E, Nicolaides NC, Stefa A, et al. Childhood obesity and leucocyte telomere length. European journal of clinical investigation. 2019;49(12):e13178.

15. Ludlow AT, Lima LC, Wang J, Hanson ED, Guth LM, Spangenburg EE, et al. Exercise alters mRNA expression of telomere-repeat binding factor 1 in skeletal muscle via p38 MAPK. Journal of applied physiology. 2012;113(11):1737-46.
